# Phenotypic variability in a French family with a novel mutation in the *BEST1* gene causing multifocal best vitelliform macular dystrophy

**Published:** 2011-01-29

**Authors:** Emmanuelle Lacassagne, Aurore Dhuez, Florence Rigaudière, Anouk Dansault, Christelle Vêtu, Karine Bigot, Véronique Vieira, Bernard Puech, Sabine Defoort-Dhellemmes, Marc Abitbol

**Affiliations:** 1 Université Paris-Descartes, Faculté de Médecine Paris Descartes - site Necker, CERTO, Paris, France; 2 Service d’Explorations fonctionnelles Visuelles, Hôpital Lariboisière, AP-HP, Paris, France; 3 CHRU de Lille, Hôpital Roger Salengro, Service d’Explorations fonctionnelles de la Vision, Lille, France; 4 AP-HP, Service d’Ophtalmologie du CHU Necker-Enfants Malades, Paris, France

## Abstract

**Aims:**

To describe genetic and clinical findings in a French family affected by best vitelliform macular dystrophy (BVMD).

**Methods:**

We screened eight at-risk members of a family, including a BVMD-affected proband, by direct sequencing of 11 bestrophin-1 (*BEST1*) exons. Individuals underwent ophthalmic examination and autofluorescent fundus imaging, indocyanine green angiography, electro-oculogram (EOG), electroretinogram (ERG), multifocal ERG, optical coherence tomography (OCT), and where possible, spectral domain OCT.

**Results:**

The sequence analysis of the *BEST1* gene revealed one previously unknown mutation, c.15C>A (p.Y5X), in two family members and one recently described mutation, c.430A>G (p.S144G), in five family members. Fundus examination and electrophysiological responses provided no evidence of the disease in the patient carrying only the p.Y5X mutation. Three patients with the p.S144G mutation did not show any preclinical sign of BVMD except altered EOGs. Two individuals of the family exhibited a particularly severe phenotype of multifocal BVMD—one individual carrying the p.S144G mutation heterozygously and one individual harboring both *BEST1* mutations (p.S144G inherited from his mother and p.Y5X from his father). Both of these family members had multifocal vitelliform autofluorescent lesions combined with abnormal EOG, and the spectral domain OCT displayed a serous retinal detachment. In addition, ERGs demonstrated widespread retinal degeneration and multifocal ERGs showed a reduction in the central retina function, which could be correlated with the decreased visual acuity and visual field scotomas.

**Conclusions:**

A thorough clinical evaluation found no pathological phenotype in the patient carrying the isolated p.Y5X mutation. The patients carrying the p.S144G variation in the protein exhibited considerable intrafamilial phenotypic variability. Two young affected patients in this family exhibited an early onset, severe, multifocal BVMD with a diffuse distribution of autofluorescent deposits throughout the retina and rapid evolution toward the loss of central vision. The other genetically affected relatives had only abnormal EOGs and displayed no or extremely slow electrophysiological evolution.

## Introduction

Best vitelliform macular dystrophy (BVMD) [1] is one of the most frequent form of autosomal dominant macular dystrophy. It is associated with mutations in the bestrophin 1 gene (*BEST1*) [2,3] and results from dysfunction of the retinal pigment epithelium (RPE). Mutations in the *BEST1* gene are detected in nearly all BVMD cases with a positive family history. Cases reported as BVMD without *BEST1* mutations have no family history of the disease and may have either been misdiagnosed or may represent phenocopies [4-6]. The *BEST1* gene is on chromosome 11q12 (NM_004183), spans 15 kb of genomic DNA, and contains 11 exons of which ten are protein coding [2,3]. The gene encodes a protein of 585 amino acids called bestrophin-1 (BEST-1 protein) [7], predominantly expressed in the basolateral plasma membrane of the RPE. The *BEST1* gene is the founding member of a family of four paralogs; the other three are called *BEST2*, *BEST3*, and *BEST4* [8]. There is various evidence that BEST-1 protein, as assessed in overexpression experiments, functions as a Cl^-^ channel influenced by [Ca^2+^]i [9] and that human BEST-1 protein is also highly permeable to HCO_3_^-^, indicating that human BEST-1 protein may also function as an HCO_3_^-^ channel [10]. Alterations of the BEST-1 channel caused by *BEST1* gene mutations may account for the diminished light peak–dark trough ratio (Arden ratio typically ≤150%) of the electro-oculogram (EOG) [11,12], which is characteristic of BVMD and associated in most cases with a normal full-field electroretinogram (ERG). An abnormal EOG has been considered essential for a diagnosis of BVMD in patients with vitelliform lesions detected by fundoscopy. Although a large majority of BVMD patients meets this criterion, several studies indicate that the EOG may initially be normal or even remains normal in *BEST1* mutation carriers, even in those who are clinically affected [13]. Multifocal ERG (mfERG) findings [14] for most BVMD patients are abnormal.

Several classifications of BVMD based on the aspect of fundus have been proposed. The most frequently cited classifications [11,15,16] are those by Deutman, Mohler, and Gass. In its initial stages, the deposits resemble an egg yolk (vitelliform stage), but later the vitelliform lesions disperse like “scrambled egg” (vitelliruptive stage). The next stage involves the formation of pseudohypopyon lesions in which the affected area becomes deeply and irregularly pigmented due to the accumulation of yellowish autofluorescent material in the subretinal space. The final atrophic stage has the appearance of scarring, sometimes associated with choroidal neovascularization. Although the classifications generally agree, the evolution of BMVD stage by stage described by these classifications is not always followed. The stages do not always occur consecutively or even inevitably in all patients. Many BVMD lesions simultaneously show characteristics of different BVMD stages [1]. Indeed, there is a substantial clinical heterogeneity in classical BVMD, including variable presentation and unpredictable course. The considerable variability of ophthalmoscopic lesions can make a clinical diagnosis of BVMD challenging. The disease shows an irregular mode of inheritance with highly variable expressivity [17] within families and even between eyes of an affected individual.

The clinical spectrum has recently been enlarged [18] by the description of atypical forms of BVMD in patients with a *BEST1* mutation. These forms include lesions simulating pattern dystrophy with a mildly reduced EOG Arden ratio, multifocal vitelliform macular dystrophy with an absent light peak on EOG, and discrete RPE changes in the fovea.

Here, we report the clinical features of eight at-risk members of a French family; two young members of this family have multifocal BVMD. We identified two novel nucleotide mutations in the *BEST1* gene. Clinical ophthalmic investigations, including fundus autofluorescence, indocyanine green angiography (ICG), optical coherence tomography (OCT), full-field ERGs, and mfERGs, of these individuals confirm the incomplete penetrance and the highly variable expression of *BEST1* mutations between affected individuals of the same family.

## Methods

### Patients

**Figure 1:**
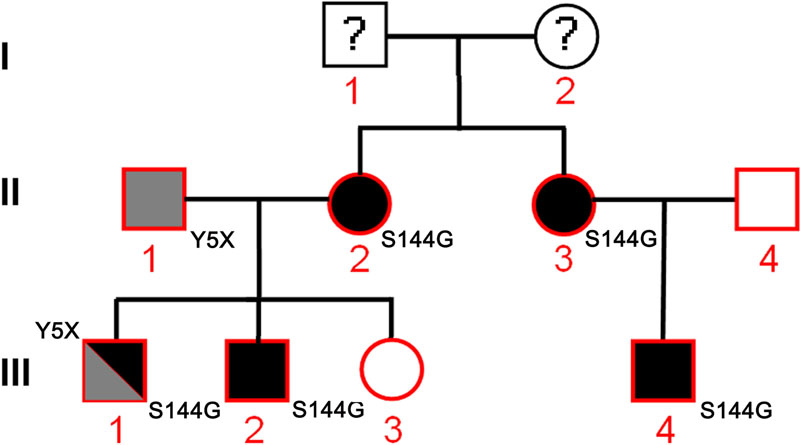
Pedigree of the family studied and segregation of the *BEST1* mutant alleles. This figure shows the pedigree of a french family displaying an unusual phenotype reminiscent of very atypic bestrophinopathy. The clinical status of I-1 and I-2 are unknown. The red shapes denote genotyped individuals. White circles represent unaffected females, filled circles affected females, white squares represent unaffected males and filled squares affected males. The p.Y5X mutation is shown in gray and the p.S144G mutation is shown in black. Individual III-1 (the proband) harbors both mutations.

We performed a genetic analysis and a complete ophthalmological examination of eight members of a single family (Figure 1), one of whom was diagnosed with BVMD. The members of this family were recruited from the north and the east of France as well as from Ile de France. They were not affected by any extra-retinal disease. The proband, patient III-1, a boy, was born in 1987 as the first of three children, and the onset of visual symptoms was diagnosed at the age of 6 years. At 20 years of age, the symptoms started to severely affect his everyday activities. Molecular screening of the *BEST1* gene and clinical examination were performed in the proband, his parents (II-1, 48 years old is the father; II-2, 44 years old is the mother), his 19-year-old brother (III-2) and his 16-year-old sister (III-3), his aunt (II-3, 33 years old) and his uncle (II-4, 41 years old), and his 9-year-old cousin (III-4).

### Clinical examination

All family members underwent standard methods of ophthalmic examination (interrogation of the patients, best-corrected visual acuity, study of the lacrimal film, slit-lamp examination, measurement of the intraocular pressure, study of color vision, study of the visual field by the Humphrey® Field Analyzer which is one of the world’s most widely used perimeter (Carl Zeiss Meditec France SAS, Le Pecq), manual fundoscopy, and digital fundoscopy imaging obtained with the Topcon SL-D7 slit lamp (Topcon S.A.RL. Clichy, France) for each eye, autofluorescent imaging analysis, OCT imaging analysis, ICG, and an electrophysiological examination as described below. The slit-lamp examinations were performed with the latest biomicroscope produced by HAAG-STREIT (Chambery, France). The digital imaging of the ocular fundus was performed by the latest digital TOPCON biomicroscope available (TOPCON SLD7) equipped with a high-resolution camera (TOPCON SARL, Clichy, France). The autofluorescent imaging ICG, and OCT scans were in most cases performed with the SPECTRALIS HRA-OCT combined with the optical coherent tomography module device - produced by Heidelberg Engineering- (SANOTEK, L’Hay Les roses, France). OCT3 Stratus devices (Carl Zeiss Meditec France SAS, Le Pecq) were used for some of the OCT scans. The HRA platform was the first commercial angiography system to use lasers in combination with marker dyes. Using the HRA instead of white light photography allows clinicians to capture detailed images of the blood vessel structure within the retina. Spectralis HRA+OCT is a spectral domain system, sometimes called fourier domain, which scans the retina at 40,000 scans per second, to create highly detailed images of the structure of the retina. Because the OCT and HRA images are captured simultaneously, the clinician can be sure of the exact location of the area of interest and can correlate the outer visible retina structure with the internal structure.

### *BEST1* gene analysis

The study was performed in accordance with the French and European Union bioethics laws and with the guidelines of the Declaration of Helsinki. Blood samples were collected from patients after informed consent was signed by the adults or by both parents of each child involved (under 12 years-old). The veinous blood samples were collected into EDTA tubes by specialized nurses using BD vacutainer systems (Becton-Dickinson SAS, Le Pont de Claix, France), kept at room temperature and used for DNA extraction less than 24 h after they had been collected. The blood (10 ml) was prepared in lysis buffer (100 mM Tris-HCl pH 7.5, 5 mM EDTA) and then centrifuged several times at 500 xg for 15 min at 4 °C to collect a clean pellet with blood lymphocytes. The pellet was solubilized using 0.5% Lauryl-sarcosyl (Sigma Aldrich, Lyon, France), and incubated with 1 µg of proteinase K (Invitrogen, Cergy Pontoise, France) in 5 ml lysis buffer overnight at 55 °C. The DNA was then purified by ethanol precipitation.

Each exon of the BEST1 gene was amplified from genomic DNA by PCR using the intronic oligonucleotide primers and the PCR conditions described in Table 1.

**Table 1:** Primers and PCR conditions used for amplifying each exon of the *BEST1* gene

Exon	Primers	Sequences (5′-3′)	Size	PCR Conditions
1	1F	CCGTTGCTTTGAGCAGATT	265	MgCl_2_ 2 mM, dNTP 0,125 µM, Primers 0,1 µM, DMSO 7,5%, TouchDown PCR 62–58 °C
	1R	AAGGCCTCAAAGCCCCAG		
2	2F	CAGGGCCTCTGATCCCTAC	341	MgCl_2_ 1 mM, dNTP 0,2 mM, Primers 1 µM, annealing temperature 56 °C
	2R	GTGAACTGGTACACTGGCCC		
3	3F	GGGACAGTCTCAGCC ATCTC	238	MgCl_2_ 1 mM, dNTP 0,2 mM, Primers 1 µM, annealing temperature 59,5 °C
	3R	CAGCTCCTCGTGATCCTCC		
4	4aF	CGCTCGCAGCAGAAAGCT	305	MgCl_2_ 1 mM, dNTP 0,125 mM, Primers 0,25 µM, annealing temperature 57 °C
	4aR	TGTAGACTGCGGTGCTGAG		
	4bF	GGCTTCTACGTGACGCTGGT	317	MgCl_2_ 1 mM, dNTP 0,125 mM, Primers 0,25 µM, annealing temperature 57 °C
	4bR	TCCACCCATCTTCCATTC		
5	5F	ATCCCTTCTGCAGGTTCTCC	274	MgCl_2_ 1,5 mM, dNTP 0,2 mM, Primers 0,1 µM, DMSO 5%, annealing temperature 56 °C
	5R	AAACCTTGTTTCCTGTGGACC		
6	6F	GGGCAGGTGGTGTTCAGA	181	MgCl_2_ 1 mM, dNTP 0,2 mM, Primers 1 µM, annealing temperature 58 °C
	6R	CCTTGGTCCTTCTAGCCTCAG		
7	7F	CATCCTGATTTCAGGGTT CC	266	MgCl_2_ 2 mM, dNTP 0,125 µM, Primers 0,1 µM, DMSO 7,5%, TouchDown PCR 62–58 °C
	7R	CTCTGGCCATGCCTCCAG		
8	8F	AGCTGAGGTTTAAAGGGGGA	215	MgCl_2_ 1 mM, dNTP 0,125 mM, Primers 0,25 µM, DMSO 5%, annealing temperature 56 °C
	8R	TCTCTTTGGGTCCACTTTGG		
9	9F	ACATACAAGGTCCTGCCTGG	298	MgCl_2_ 2 mM, dNTP 0,125 mM, Primers 0,1 µM TouchDown PCR 62–58 °C
	9R	GCATTAACTAGTGCTATTCTAAGTTCC		
10	10aF	GGTGTTGGTCCTTTGTCCAC	591	MgCl_2_ 1,5 mM, dNTP 0,125 mM, Primers 0,25 µM, DMSO 5%, TouchDown PCR 62–58 °C
	10aR	CTCTGGCATATCCTCAGGT		
	10bF	CTTCAAGTCTGCCCCACTGT	457	MgCl_2_ 1,5 mM, dNTP 0,125 mM, Primers 0,25 µM, DMSO 5%, TouchDown PCR 62–58 °C
	10bR	TAGGCTCAGAGCAAGGGAAG		
11	11F	CATTTTGGTATTTGAAATGAAGG	216	MgCl_2_ 1,5 mM, dNTP 0,125 mM, Primers 0,25 µM, annealing temperature 54 °C
	11R	CCATTTGATTCAGGCTGTTGG		

For each pair of primers, the amplicon size is indicated in base pairs. F: forward primer; R: reverse primer. Exons 4 and 10 were amplified as two overlapping fragments. In each case, primer pair aF and aR amplifies the upstream 5′ part of the target genomic DNA, and primer pair bF and bR amplifies the downstream 3′ part of the target genomic DNA encompassing the specific exon sequences.

Each PCR was performed in a reaction volume of 60 μl containing 150 ng of patient genomic DNA as a template, 10 mM Tris-HCl at pH 8.3, flanking primers, MgCl_2_, dNTP, DMSO, and 0.4 U of *Taq* DNA polymerase (Invitrogen, Cergy Pontoise, France). PCR was performed in a PTC 225 automated thermal cycler (MJ Research, Waltham, MA). Amplified fragments were purified using the Concert kit with NucleoFast 96-well PCR plates (Macherey-Nagel, Hoerdt, France) and then analyzed by direct sequencing using an ABI PRISM 3100 DNA sequencer (Applied Biosystems, Courtaboeuf, France).

A control group was constituted from 100 unrelated individuals from France who were unaffected by any form of macular degeneration or inherited retinal dystrophy and with no family history of BMVD. These controls served to ensure that the mutations identified were not simply common polymorphisms.

### Information retrieved from DNA and protein databases

Genomic DNA and cDNA sequences of the human *BEST1* gene are available at the GenBank database (NM_004183).

Using the multiple alignment program CLUSTALW, protein sequences were aligned with the reference sequence NP_004174. Nucleotide variations leading to unchanged amino acids in the protein sequence were analyzed using ESE finder to determine whether these nucleotidic changes had any effect on mRNA splicing. A check was performed at the Regensburg University database Website to verify whether the nucleotide sequence variants of the *BEST1* gene that we found had already been submitted to the database.

### Electrophysiological testing

The electroretinograms (flash ERG, mfERG) and EOG [19,20] were obtained during routine clinical examination in accordance with the standards of the International Society for Clinical Electrophysiology of Vision. The EOG was recorded first, followed by flash ERG and mfERG. Stimulus and data acquisition were controlled with a Moniteur Ophtalmologique system (Métrovision, Lille, France). The pupils were fully dilated before the three tests (Tropicamide [CIBA-vision, Blagnac, France] 2 mg/0.4 ml instilled 30 min before test recordings). The EOG mainly included the response of the basolateral membrane of the RPE. Flash ERG included the scotopic responses (rod and mixed response) and the photopic responses (photopic-oscillary potentials, cone and flicker responses). MfERG included the photopic responses of 61 hexagons distributed into five concentric rings on the posterior pole (40°). The central response corresponded to the fovea.

### Data analysis

Amplitudes and implicit times of a- and b-flash-ERG waves were compared to those measured in normal subjects. N1-P1 and N2-P2 summated amplitudes of each mfERG ring were measured and compared to normal values. Data falling within plus or minus two standard deviations (SDs) of the mean for normal subjects were considered as normal. Response amplitudes superior to the mean plus two SDs or inferior to the mean minus two SDs were considered to be abnormally high or decreased, respectively. The EOG Arden ratio is the light peak as a percentage of the dark trough and was calculated automatically. Patients displaying an Arden ratio below 150% are usually considered as affected patients.

## Results

### Genetic analysis

**Figure 2:**
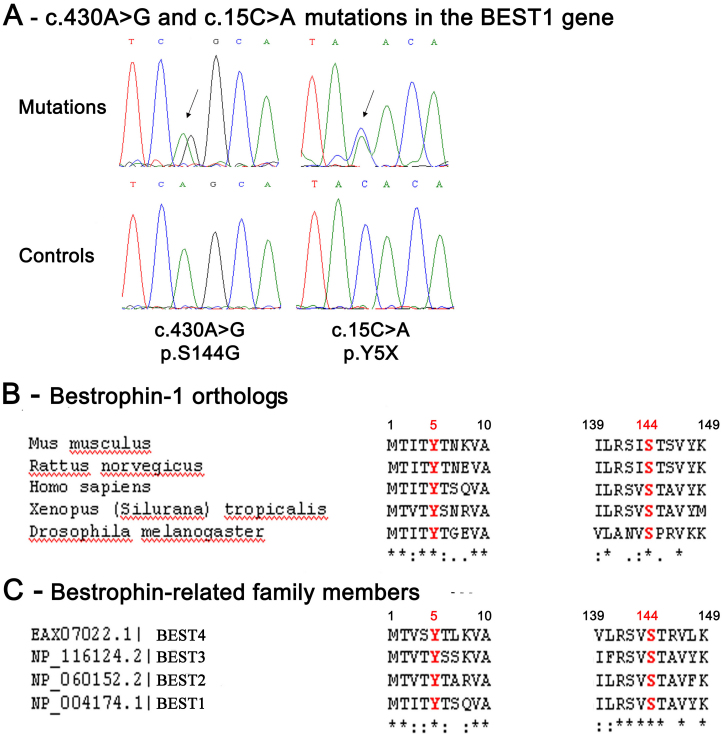
Two novel nucleotidic mutations in the *BEST1* gene. Electrophoregrams of the *BEST1* gene mutations found in the affected members of the French family studied and phylogenetic conservation throughout evolution of the normal BEST-1 amino-acid residues affected by these mutations. **A**: These electrophoregrams show heterozygous mutated nucleotides in the *BEST1* gene: An adenine (A) is replaced by a guanine (G) at the 430th nucleotidic position of the *BEST1* cDNA sequence (c.430A>G) and and a cytosine (C) is replaced by an adenine (A) at the 15th nucleotidic position of the *BEST1* cDNA sequence (c.15C>A) (top panel), and normal sequences (low panel). The peaks in red indicate thymidine (T), green indicate A, black indicate G, and blue indicate C. **B**: This panel shows the multiple sequence alignment of human bestrophin-1 protein (BEST-1 protein; NP_004174) with the BEST-1 protein sequences from *Mus musculus* (NP_036043.2), *Rattus norvegicus* (NP_001011940.1), *Xenopus tropicalis* (BAH70274.1), and *Drosophila melanogaster* (AAF54503.1). This multiple sequence alignment highlights the strong conservation throughout evolution of the amino-acid residues of the normal BEST-1 protein which were found affected by mutations in this study. **C**: This panel shows the multiple sequence alignment of the human BEST1 protein with the bestrophin paralogs: BEST2, BEST3, and BEST4. Alignments are zoomed into the relevant region. The amino- acids affected by a mutation are shown in red. The stars indicate 100% conservation.

Patients II-2, II-3, III-2, and III-4 (Figure 1) all carried the same, previously unreported, heterozygous nucleotide mutation, located in exon 4 of the genomic sequence (Figure 2A): an A to G substitution at position 430 of the *BEST1* coding sequence (c.430A>G). The codon AGC was thus transformed into the codon GGC, which corresponds to a serine to glycine substitution at position 144 of BEST-1 protein (p.S144G). The mutation is located between the second (TM2) and the third putative transmembrane (TM3) domain of BEST-1 protein, a region displaying good conservation in phylogenetically distant orthologs of bestrophin (Figure 2B). Furthermore, this position is completely conserved in human bestrophin-related family members (BEST2, BEST3, and BEST4; Figure 2C). The p.S144G mutation is thus in a BEST-1 protein region that has not been previously considered as a hotspot, despite harboring many mutations. A different nucleotide mutation, c.431G>A, affecting the same codon and resulting in the same amino acid substitution (p.S144G) [21] has recently been shown to cause typical monofocal BVMD in a Chinese family.

We also detected a heterozygous c.15C>A mutation (Figure 2A) in the *BEST1* gene in exon 2 in one patient (II-1), resulting in a premature stop codon instead of a tyrosine at the fifth codon (p.Y5X). The tyrosine residue at this position is highly conserved in phylogenetically distant orthologs and also in bestrophin-related family members (BEST2, BEST3, and BEST4; Figure 2B,C).

One of the eight related subjects enrolled in this study was the proband (III-1). We found that he had inherited both *BEST1* mutations, c.430A>G (p.S144G) from his mother (II-2) and c.15C>A (p.Y5X) from his father (II-1). Neither of these two mutations were found in any of the 100 unaffected controls.

### Clinical results

**Figure 3:**
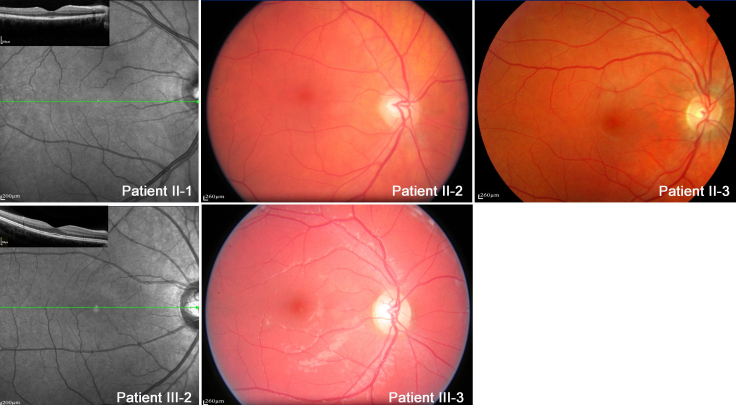
Color fundus and autofluorescent fundus/optical coherence tomography scans in patients II-1, II-2, II-3, III-2, and III-3. No macular lesion was detectable by autofluorescence imaging or optical coherence tomography scanning for patients II-1 or III-2. No macular lesion was detectable in the color fundus for patients II-2, II-3, or III-3.

II-1, II-2, II-3, III-2, and III-3 had completely normal ophthalmic examinations (Table 2, Figure 3), with normal visual acuity (200/200 for each eye) and binocularly, normal fundi, and normal OCT.

**Table 2:** Clinical presentation, electrophysiological findings, and OCTs of the patients involved in this study.

**Patient**	**Exon**	**Nucleotide Change**	**Amino Acid Change**	**Effect**	**Age of onset**	**Age at examination**	**Visual acuity at examination**	**EOG (Arden ratio: Right Eye/Left Eye)**	**Fundus examination**	**OCT**	**Flash ERGs**	**mfERG**
II-1	2	c.15C>A	p.Y5X	Stop mutation		48	0.200/200.	315/286	Normal	Normal	Normal	Normal
II-2	4	c.430A>G	p.S144G	Charge/Polarity		44	0.200/200.	145/138	Normal	Normal	Normal	Normal
II-3	4	c.430A>G	p.S144G	Charge/Polarity		33	0.200/200.	145/198	Normal	Normal	Normal	Normal
II-4		no mutation				41	0.200/200.					
III-1	2,4	c.15C>A c.430A>G	p.Y5X p.S144G	Stop mutation Charge/Polarity	6	23	20/200 (right eye) 130/200 (left eye)	129/111	Typical vitelliform lesions in both eyes	Disruption of the photoreceptor layer / Highly reflective materiel at the RPE layer in both eyes	Dramatic decrease of the ERGs responses	Retinal electrogenesis of the central cones severely altered
III-2	4	c.430A>G	p.S144G	Charge/Polarity		19	0.200/200	124/130	Normal	Normal	Normal	Normal
III-3		no mutation				16	0.200/200.					
III-4	4	c.430A>G	p.S144G	Charge/Polarity	4	9	30/200 (right eye) 200/200 (left eye)	263/156	Typical (right eye) or faded (left eye) vitelliform lesions	Disruption of the photoreceptor layer (right eye), abnormal thickness of the foveal region (left eye)	Dramatic decrease of the ERGs responses	

**Figure 4:**
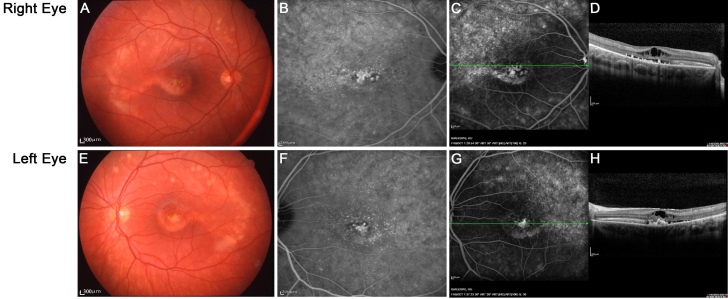
Right and left eye color fundus, autofluorescent fundus, indocyanine green angiography, and optical coherence tomography scans in the proband (patient III-1). Well demarcated vitelliform lesions in the central macula are detected by fundoscopy (**A**-**E**) and are also apparent on the autofluorescence image (**B**-**F**) and indocyanine green angiography (**C**-**G**) in both eyes. Optical coherence tomography images through the fovea show a highly reflective thickened layer at the level of the retinal pigment epithelium and choriocapillaris of both eyes and well circumscribed elevation of the retinal pigment epithelium in both eyes (**D**-**H**).

III-1 is the proband. He was 23 years old with no known familial history of ocular disease at diagnosis of BVMD at the age of 6 years. His best-corrected visual acuity is 20/200 in the right eye and 130/200 in the left eye. Fundus examination showed vitelliruptive lesions with a scrambled egg appearance and dispersion of the vitelliform material but no sign of atrophy (Figure 4A,E) confirmed by autofluorescence imaging (Figure 4B,F). The ICG detected a hypofluorescence at the early stages of the angiographic sequence in both eyes (Figure 4C,G) and subsequently abnormal hyperfluorescence at the late stages. Fluorescein angiography revealed significant early hyperfluorescence that increased in intensity at the late stage of the angiographic sequence in both eyes and was associated with moderate leakage (data not shown). Spectral domain OCT scans showed striking abnormalities (Figure 4D,H) with the absence of the foveal pit, serous retinal detachment, and cystoid macular edema and interruption of the outer limiting membrane. The deep retinal layers were irregular, with an abnormal junction between the inner and the outer segments, multiple hyper-reflective foci, and deep material deposits on the pigment epithelial layer.

**Figure 5:**
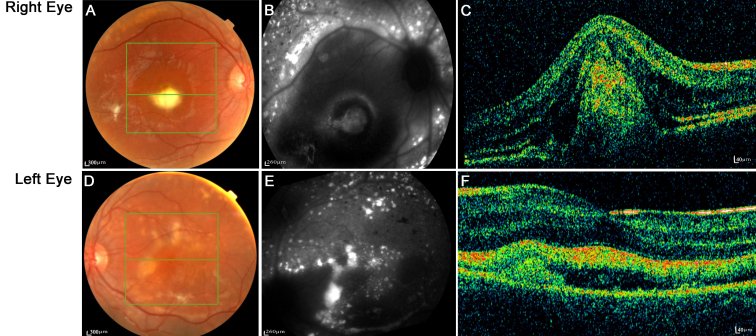
Fundoscopy, autofluorescence and optical coherence tomography (OCT3) imaging of a severely affected patient (III-4). Typical vitelliform lesions are visible on the ophthalmoscopic appearance of the right eye (**A**, **B**, **C**) and left eye shows fragmented vitelliform lesions (**D**, **E**, **F**). Green lines indicate abrupt transitions and the frame of the fundus that was scanned by optical coherent tomography (OCT). The middle green lines of (**A**, **D**) indicate the horizontal axis of the OCT scan shown in (**C**, **F**).

III-4 is the proband’s first cousin. He was 9 years old on inclusion in this study and he had been diagnosed as having multifocal BVMD at the age of 4 years. Right eye ocular fundoscopy showed (Figure 5A) a major macular yellow lesion associated with multiple smaller more peripheral vitelliform foci in the vitreous cavity. This lesion was also apparent on autofluorescence images (Figure 5B), corresponding to the classic appearance of the vitelliruptive stage. OCT along a central horizontal axis of the same eye revealed a highly reflective area (Figure 5C,D) corresponding to a prominent mass of heterogenous material emerging from the choroid and disrupting the RPE completely, pushing the central photoreceptor layer toward the vitreous cavity. The macula of the left eye was surrounded by yellow deposits (Figure 5E,F) but was devoid of any central vitelliform disc. Nevertheless, OCT revealed (Figure 5G,H) that the foveal region was abnormally thick due to abnormal neuroretinal detachment from the RPE in the region. This detachment had probably been triggered by an abnormal accumulation of fluid within the choriocapillaris and between the RPE and the fovea. This serous detachment had already caused displacement, without any significant disorganization, of the photoreceptor layer. The visual acuity of the left eye was preserved, whereas that of the right eye had progressively declined since early childhood to 30/200. The decline in the visual acuity of the right eye was rigorously evaluated and corresponded to the lesions detected by fundoscopy, autofluorescence imaging, and OCT and especially to the complete disruption of the RPE and the prominent disorganization of the neuroretinal layers in the macular region. The preservation of the visual acuity of the left eye was consistent with the central ocular fundoscopic aspect that showed the absence of a central macular vitelliform disk in association with multiple small diffuse vitelliform lesions throughout the retina. These lesions were especially dense and numerous around the foveal and macular regions. Despite the existence of a subretinal edema in the foveal region, numerous abnormal RPE abnormalities throughout the retina, the existence of multiple vitelliform autofluorescent lesions outside the foveal area, the relative preservation of the outer retinal structure in the foveal region, combined with a normal organization of all the inner layers in a large part of the macular region explains the relative preservation of the visual acuity of the left eye.

### Electrophysiological results

Two of the eight family members declined to undergo electrophysiological examinations, and consequently only the following six were examined: II-1 (proband’s father), II-2 (proband’s mother), II-3 (proband’s aunt), the proband III-1, III-2 (proband’s young brother), and III-4 (proband’s young cousin; see Figure 1).

**Figure 6:**
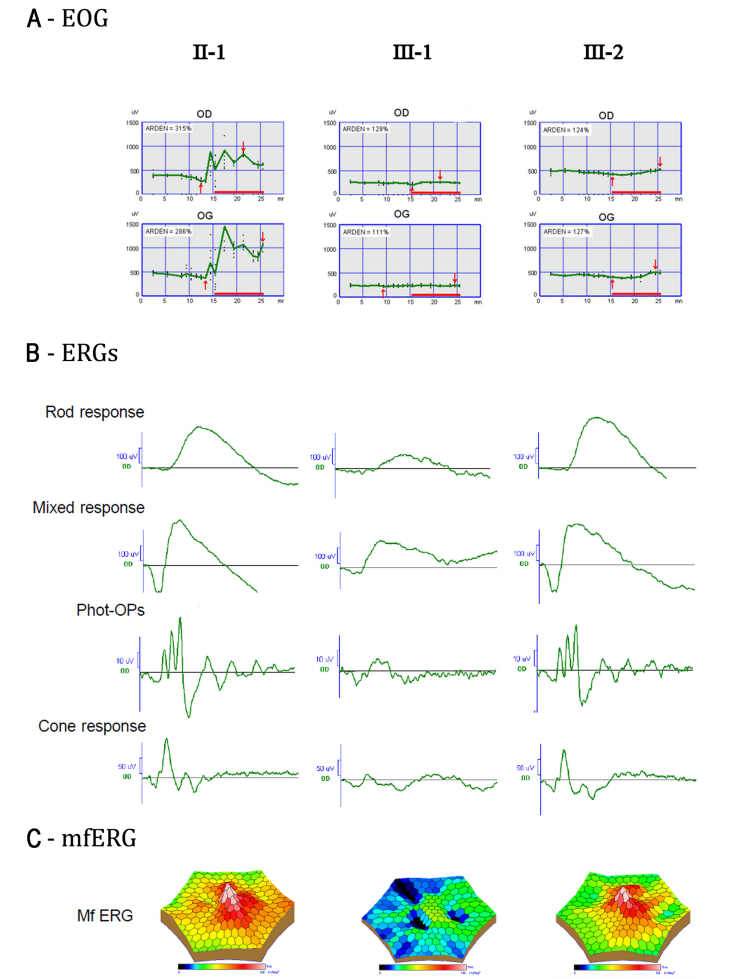
Electrophysiology measurements of three representative cases of the family studied. This figure represents electro-oculograms (EOGs; **A**), right eye flash electroretinograms (ERGs; **B**) and right eye multifocal electroretinograms (mfERGs; **C**) in patients carrying one mutation heterozygously (II-1: p.Y5X; III-2: p.S144G) or both mutations (III-1). Findings are based on ISCEV standard. Patients II-2 and II-3 displayed electrophysiological findings similar to III-2 and patient III-4 displayed electrophysiological findings similar to III-1. Except for II-1, the amplitudes for the light phase of the EOG (**A**) were abnormal with a reduction in the Arden ratio (EOG light rise <150%). In patient III-1 (and III-4), flash ERGs show generalized decreased rod and cone photoreceptor amplitudes and decreased photopic oscillatory potentials amplitude (Phot-Ops; **B**). MfERG records a reduced central function with relative preservation of the amplitude response and timing from the surrounding macula (**C**).

The electrophysiological results for patients II-1, III-1, and III-2 are shown in Figure 6, and those for patients II-2 and II-3 were similar to III-2 (data not shown).

The EOG of II-1 was normal (Arden ratio ≥150%). Despite being clinically normal, EOGs (Figure 6A) for II-2, II-3, and III-2 were flat with no light peak rise. Flash ERGs (Figure 6B) for II-1, II-2, II-3, and III-2, which reflect rod and cone function, were normal (scotopic and photopic responses). MfERGs (Figure 6C), which reflect the function in the macular region, were within normal limits for all four of these patients.

The proband III-1 and his cousin III-4 showed no EOG light peak rise (“flat” EOG) in both eyes (Figure 6A), indicating a disorder between the photoreceptor layer of the outer retina and the RPE. Flash ERGs for III-1 showed (Figure 6B) a significant decrease in scotopic and photopic response amplitudes. Similarly, mfERGs (response of the posterior fundus) for III-1 showed (Figure 6C) a significant decrease in the amplitude of the foveal response for each eye, although the peripheral mfERG amplitudes were within normal limits. Decreased mfERG amplitudes,  reflecting the macular dysfunction associated with the macular oedema, very well detected by OCT imaging, and loss of integrity of the foveal photoreceptor inner/outer segment junction correlated with decreased vision. MfERGs could not be performed for III-4, but he displayed low flash ERGs, similar to III-1 (data not shown).

In summary, examinations of this family (Table 2), found unremarkable fundi but altered EOGs with low Arden ratios for the proband’s mother (II-2), young brother (III-2), and aunt (II-3). Findings for II-2, II-3, and III-2 are consistent with the early stages of BVMD (probably still in the previtelliform stage), although these individuals did not suffer any symptomatic visual handicap at the time of examination. It is possible that they will not develop clinical manifestations of BVMD during their life if the pathogenic allele that they carry is not clinically penetrant.

Patients III-1 and III-4, who presented with visual failure and macular degeneration since childhood, exhibited a phenotype consistent with bestrophinopathy (EOG, OCT), probably at the vitelliruptive stage, associated with multiple vitelliform autofluorescent foci localized outside the foveal region (mostly around the macular region and in the peripheral retina). However, the flash ERG and mfERG for III-1 indicated a diffuse altered RPE–neuroretina junction and a generalized effect on cone and rod function with a predominance of the functional alterations in the foveolar region.

## Discussion

We report an analysis of *BEST1* gene mutations of eight subjects from a French family in which initially only the proband III-1 was known to be affected by early onset multifocal BVMD. Genetic analysis identified two mutations — p.Y5X and p.S144G — one of which, p.S144G, co-segregates with numerous characteristics of the clinical spectrum of BVMD and appears to be the pathogenic mutation [22]. We studied the different phenotypes in detail by performing repeated clinical examinations and integrated imaging analysis with recently available equipment (fundus photography, fundus autofluorescent photography, ICG, OCT scans) and electrophysiological tests (ERGs, EOG) of the family members who consented to clinical and genetic investigations. Our study and a recent description of a Danish family show the importance of combining thorough and repeated clinical examinations involving the use of the latest methods available for exploring the retinal function with rigorous molecular genetics analysis to address the complexity of BVMD.

A large number of allelic variants of the *BEST1* gene have been identified and are recorded in the Regensburg University database. Missense mutations comprise 92% of the mutations [6], and only five null *BEST1* mutations have been reported, three of which have been associated with a BVMD phenotype. Two of these mutations, p.P260fsX288 [23] and p.H490fsX514 [24], were identified as heterozygous in isolated BVMD cases with no information available about any of the family members and no mutation identified on the counter allele. The third mutation, p.Y29X, was detected in a Swedish family with a rare *BEST1* genotype in which two sisters, aged 30 and 33, were compound heterozygous for a missense mutation and a null mutation and presented with vitelliform dystrophy and electrophysiological signs of widespread retinal degeneration [22]. This phenotype shares similarities with that of the proband (patient III-1) of our French family. In addition, p.R200X [25] was found to cause ARB. In two AMD patients the p.L149X [5] mutation was identified heterozygously.

We found a novel c.15C>A mutation (p.Y5X) in the *BEST1* gene that gave rise to a truncated BEST-1 protein. Patient II-1 (the father of the proband III-1) had inherited the p.Y5X mutation heterozygously and had a normal-appearing fundus, a completely normal OCT in each retina, and even normal findings by EOG and other electrophysiological tests. Consequently, II-1 appears to have neuroretina and RPE with completely normal functioning. Therefore, one single normal copy of the *BEST1* gene might be sufficient to confer a normal phenotype. The phenotype of II-1 is that of an asymptomatic carrier.

Both missense and truncating mutations can provoke a disease phenotype by a haploinsufficiency [26] mechanism, as exemplified by the mutations causing aniridia. Most of the investigations studying function of the mutant BEST-1 protein [27-29] reported a loss of function mechanism. However, if the BVMD phenotype was caused exclusively by haploinsufficiency [21], many more truncating mutations should be observed. Neither our report nor any other report in which BVMD-affected patients and all their family members have been carefully studied clinically and molecularly provides any support for a universal haploinsufficiency mechanism. The observed BVMD phenotype of most clinically affected individuals apparently carrying heterozygous null mutations in *BEST1* [22,23] probably correspond to compound heterozygotes who carry a missense mutation on the counter allele overlooked during the first genomic screening.

Nevertheless, given that vitelliform macular dystrophy appears with incomplete penetrance and variable age onset [30], we cannot exclude the possibility that II-1 may subsequently develop a late onset form of BVMD, although this appears unlikely from the thorough and accurate data we have for this family.

In this report we also describe five related patients carrying a novel heterozygous c.430A>G transition in the *BEST1* gene, which leads to a p.S144G substitution in the BEST-1 protein. This substitution replaces a noncharged polar amino acid (serine) with a small nonpolar or apolar amino acid (glycine). The serine at position 144 is an invariant residue in human bestrophin-related family members and is highly conserved in other species, suggesting an important functional or structural role of this amino acid in normal BEST-1 protein. The p.S144G substitution may therefore affect the functioning of the BEST-1 calcium-activated chloride channel. However, rather than causing a severe phenotype, the p.S144G mutation segregates with a phenotype that varies from generation to generation for probably more than two generations. The reduced light peak on the EOG was a completely penetrant electrophysiological manifestation. BEST-1 calcium-activated chloride channels are in the RPE basolateral membrane and contribute to the generation of EOG light peak. Thus, the substitution in the BEST1 protein probably modifies the function of these channels and provokes an abnormal electrophysiological coupling between the RPE and the neuroretina (EOG). Alternatively, the coupling may still exist, whereas the intracellular downstream signaling pathways of the *BEST1*-mutated channels might be altered. Three family members of the family were found to be carriers of this BEST1 mutation without any clinical expression of the disease except an abnormal EOG. Two family members exhibited characteristic lesions of multifocal BVMD. We report an early onset and fast-evolving form of multifocal BVMD first in the proband (III-1), before any family clinical history was known, and then in his younger first cousin (III-4).

The proband was compound heterozygous for mutations in the *BEST1* gene, carrying the p.Y5X mutation transmitted by his father and the p.S144G mutation transmitted by his mother. This is only the second report [22], as far as we are aware, of a patient who is compound heterozygous for the *BEST1* gene. As with most BVMD patients, mfERG for III-1 was abnormal. However, patient III-4, carrying solely the p.S144G mutation, displayed an unexpectedly severe phenotype, extremely similar to that of III-1. Indeed, the decreased vision, marked scrambled egg appearance of the central fundus, structural and functional abnormalities of the fovea (revealed by OCT scans), abnormal EOG Arden ratio, and abnormal mfERG are typical features of BVMD.

Patients III-1 and III-4 also share abnormally low and delayed rod and cone responses, indicating widespread retinal involvement, although photopic and scotopic ERGs are generally normal in BVMD patients. The mutation p.S144G was recently described in a Chinese patient with monofocal BVMD in a study that included fundus autofluorescence, EOG, and OCT. Apparently no full-field ERGs were performed [21] for this particular patient or his relatives. Our data collected from the French family confirm a generalized RPE dysfunction associated with the p.S144G mutation, possibly suggestive of an ARB pattern. Indeed, mutations in the *BEST1* gene have been found not only in patients with BVMD [2,3] but also in patients with ARB [25] and AVMD [4] as well as in patients affected by autosomal dominant or autosomal recessive retinitis pigmentosa [31].

ARB [25] has an autosomal recessive inheritance pattern. This means that homozygosity or compound heterozygosity [25,32] is required to contribute to the severity of the ARB phenotype. In the French family, the electrodiagnostic tests (EOG) and the DNA analysis were consistent with an autosomal dominant mode of BVMD transmission. In addition the p.Y5X mutation was initially suspected to result in haplosufficiency and, as stated previously, the abnormal EOG is probably caused by the observed genotype in the heterozygous carriers of the p.S144G mutation. Although ARB exhibits some pathologic similarities to BVMD, the recessive pattern of inheritance of ARB and the distinct clinical characteristics (including the characteristic vitelliform lesions) indicate that the phenotypes observed in the French family most likely belong to the BVMD clinical spectrum. Furthermore, a low Arden ratio (≤150%) differentiates BVMD from all other bestrophinopathies [1].

The phenotypes observed in the French family studied are clearly different from most bestrophinopathies described so far. Actually, they are reminiscent of the atypic BVMD phenotypes caused by other *BEST1* mutations reported recently [18]. Finally, the abnormal ERGs recorded from the proband III-1 and his first cousin III-4 and the similar clinical, imaging, and electrophysiological data collected in these two patients can be easily reconciled with the diagnosis of BVMD. Significantly, it was previously reported that flash ERGs may decline with time during the evolution of the disease phenotype, reflecting severe rod and cone photoreceptor dysfunction [22,30]. Thus, these patients with multifocal vitelliform lesions combined with an autosomal dominant inheritance pattern, abnormal EOG findings, and a mutation in the *BEST1* gene should be diagnosed as having multifocal BVMD [12], even though there are abnormal ERGs.

Our data indicate an incomplete penetrance and a variable expressivity of a mutation in the *BEST1* gene in a single family. The EOG and the DNA analysis suggest an autosomal dominant mode of BVMD transmission, and there is clearly a highly variable phenotype associated with the p.S144G mutation. The clinical onset was late with a slow evolution in some patients (II-2, II-3, III-2), but the onset of a severe multifocal BVMD was early with a fast evolution toward the central vision loss in at least two other patients (III-1 and III-4). This single p.S144G mutation seems to cause a broad phenotypic range, including typical monofocal BVMD in a Chinese family [21], severe multifocal BVMD with early onset in two patients of this study’s French family, and isolated abnormal EOGs in other members of the same family. This observation raises the crucial issue of the differential regulation of transcription of the mutated *BEST1* alleles within the same family and between different families and highlights the complexity of monogenic diseases in general. *BEST1* mutations do not correlate with clinical severity of BVMD patients. Indeed, some patients [33] never manifest fundus changes even with genetically confirmed BVMD and an abnormal EOG. These various observations suggest that a normal eye fundus does not rule out a diagnosis of BVMD and indicate the importance of EOG and molecular analysis.

This study leads us to the conclusion that four issues are too often overlooked in the diagnosis, follow-up, and management of BVMD: 1) This disease affects, sometimes with dramatic clinical consequences, young children and may evolve quickly toward the loss of central vision. Thus, a reassessment of the age of onset of this disease would be valuable. It would also be useful to conduct studies on independent cohorts of children belonging to families known to be affected by BVMD, including rigorous follow-up of disease evolution according to the genotype, with the systematic use of the latest generation of noninvasive diagnostic devices for the study of the human retina; 2) more than 300 different *BEST1* allelic variants have been reported in BVMD, but we are far from having a comprehensive database of *BEST1* mutations causing BVMD worldwide, largely because there is no systematic whole *BEST1* gene sequencing in affected patients; 3) the collection of accurate clinical and biologic information (most, if not all, *BEST1* mutations have been detected by sequencing *BEST1* gene coding exons without always checking the co-segregation of the nucleotide variant with the disease) and electrophysiological data at the cellular level, using patch-clamp technology [29] may help establish phenotype–genotype correlations, leading to a better understanding of BVMD; 4) various investigations and approaches need to be applied in a more systematic manner, in particular illegitimate transcription with total RNA extracted from white blood cells or from lymphoblastoid cell lines of affected patients to detect intronic mutations, PCR of genomic DNA to study gene copy numbers and long- range genomic PCR detection of deletions or rearrangements of the *BEST1* gene, systematic sequencing of the regulatory regions controlling *BEST1* gene transcription, analysis of *BEST1* gene epigenetics, and functional studies of the mutant bestrophin-1 proteins in vitro and in vivo.
